# Enhancing Anti-Cancer Efficacy in Colorectal Cancer Through Cannabinoid and Sodium Pentaborate Co-Therapy

**DOI:** 10.3390/molecules31183206

**Published:** 2026-09-11

**Authors:** Büşra Yüksel, Fikrettin Şahin, Nezaket Türkel

**Affiliations:** Department of Genetics and Bioengineering, Faculty of Engineering Natural Science, Yeditepe University, Kayışdağı, Istanbul 34755, Turkey; busra.yuksel@yeditepe.edu.tr (B.Y.); fsahin@yeditepe.edu.tr (F.Ş.)

**Keywords:** colorectal cancer, cannabinoid compounds, sodium pentaborate, apoptosis, combination therapy, ferroptosis

## Abstract

Background: Colorectal cancer (CRC) is characterized by pronounced genetic and phenotypic heterogeneity, which substantially influences therapeutic response and limits the efficacy of uniform treatment strategies. Cannabinoid-derived phytochemicals and boron-based compounds have independently been reported to modulate cancer cell proliferation, survival, and redox balance. However, the extent to which these agents interact at the cellular level and whether such interactions are dependent on tumor-specific molecular contexts remains poorly defined. Methods: Sodium pentaborate (NaB) was combined with non-cytotoxic concentrations of cannabidiol (CBD) or cannabigerol (CBG) and evaluated in HCT-116 and HT-29 colorectal cancer cell lines. Cell viability and drug interactions were assessed by MTS and combination index analyses. Apoptotic responses were examined by Annexin V/PI staining, caspase-3/7 activity assays, and transcriptional profiling of apoptosis-related genes. Cell cycle dynamics and proliferation-associated markers were analyzed by flow cytometry and quantitative PCR. In parallel, ferroptosis-associated gene expression patterns were investigated to evaluate alterations in redox and iron metabolism pathways. Results: NaB–cannabinoid combinations produced divergent biological outcomes depending on cellular background. HT-29 cells exhibited dose-dependent antiproliferative responses to NaB + CBD and NaB + CBG, with synergistic interactions observed only at selected dose combinations accompanied by increased early apoptosis. In contrast, HCT-116 cells primarily responded with cell cycle arrest and transcriptional stress signaling rather than enhanced cytotoxicity. Modulation of ferroptosis-related gene expression further indicated differential redox adaptation between the two cell models. Conclusions: NaB–cannabinoid combinations elicit distinct biological outcomes in colorectal cancer cells that are strongly determined by cellular context. While HT-29 cells are selectively sensitized to combination dose level, HCT-116 cells predominantly respond through cell cycle arrest and adaptive stress-response pathway activation. These findings emphasize the necessity of context-aware combination strategies and provide a mechanistic framework for the further development of boron–cannabinoid-based therapeutic approaches in colorectal cancer.

## 1. Introduction

Colorectal cancer (CRC), characterized by the uncontrolled proliferation of cells in the colon, rectum, or appendix of the gastrointestinal tract, is among the most lethal cancer types due to its typically late-stage diagnosis [1]. As a heterogeneous malignancy, CRC ranks as the second most commonly diagnosed cancer in females and the third in males [2]. Late diagnosis, therapeutic resistance, and metastasis contribute to its high mortality rate. While conventional treatments such as surgery, chemotherapy, and radiotherapy are standard, they are often accompanied by severe side effects and limited long-term efficacy [3,4]. Thus, identifying novel, well-tolerated compounds with improved therapeutic outcomes has become a central focus of cancer research.

**Figure 1:**
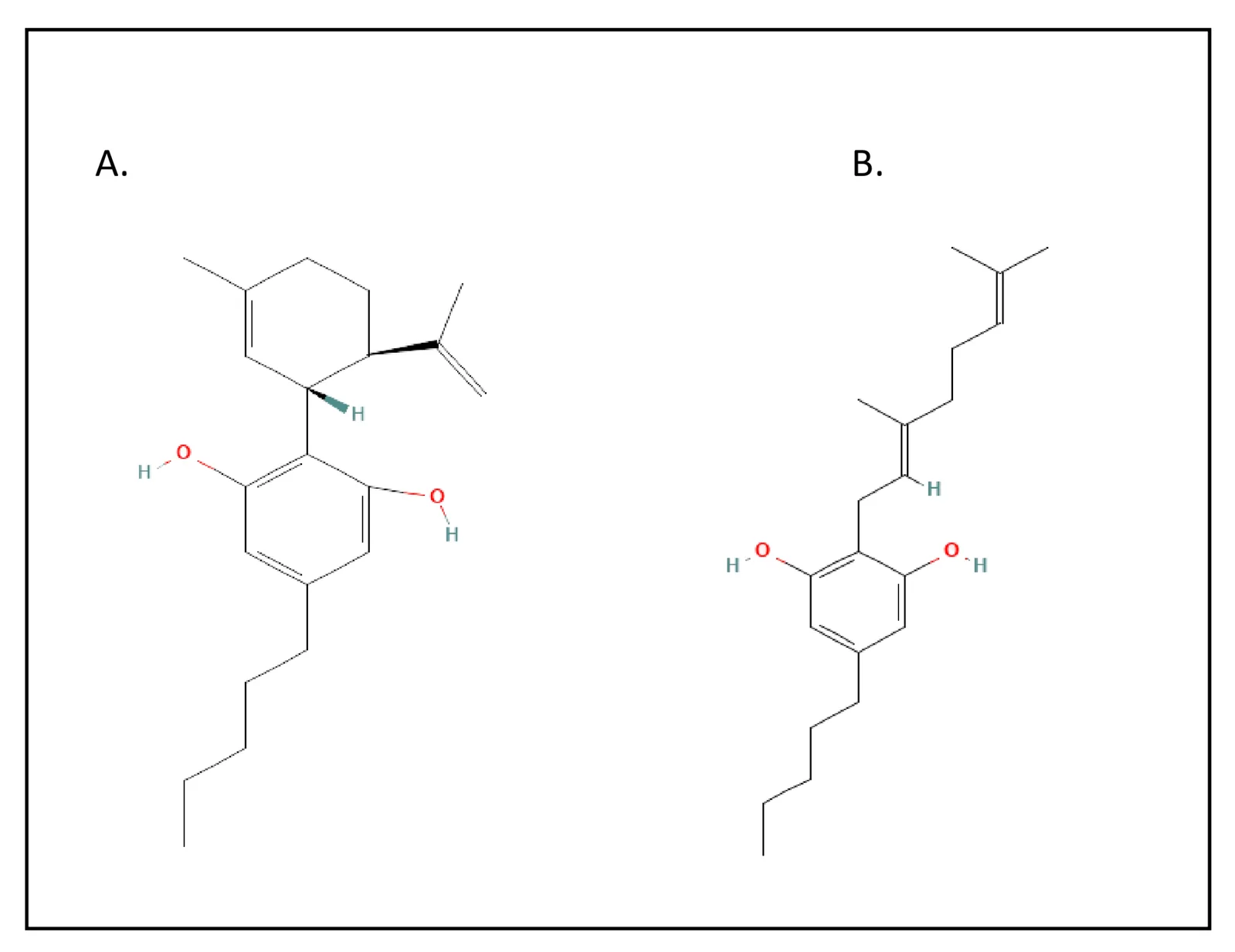
Chemical structures of the phytocannabinoids used in this study. (**A**) Cannabidiol (CBD). (**B**) Cannabigerol (CBG).

Cannabinoids, initially used to alleviate the adverse effects of cytotoxic cancer therapies—including anorexia, pain, and nausea—have more recently garnered attention for their intrinsic anti-tumorigenic properties across various cancer types [5,6]. These compounds exert growth-inhibitory, pro-apoptotic, and anti-metastatic effects by modulating diverse protein kinase pathways [6]. Over 60 cannabinoid compounds, including those derived from Cannabis sativa—such as tetrahydrocannabinol (THC), cannabidiol (CBD), cannabinol (CBN), cannabichromene (CBC), and cannabigerol (CBG)—have been extensively studied [7,8]. The chemical structures of cannabidiol (CBD) and cannabigerol (CBG), the phytocannabinoids investigated in the present study, are shown in Figure 1. Among them, CBD has demonstrated efficacy in suppressing the growth of glioblastoma, breast, lung, prostate, and colon cancers [9]. Synthetic cannabinoids like WIN also exhibit strong anti-cancer properties; both WIN and CBD induce apoptosis and inhibit proliferation in CRC and pancreatic cancer models [10]. Similarly, THC disrupts survival signaling and induces apoptosis in CRC cells, while CBG promotes reactive oxygen species (ROS) production and impedes tumor growth in vivo [11,12]. Importantly, these cannabis-derived compounds display selective cytotoxicity, affecting colon carcinoma cells while sparing normal colon tissue [13].

Boron, a nonmetallic element essential for plants, animals, and humans, is gaining attention as a pharmacologically active agent due to its versatile chemical properties [14]. Its ability to alternate between sp^2^ and sp^3^ hybridization states enables it to form stable covalent bonds, making it a valuable scaffold in drug development [15]. The U.S. FDA has approved several boron-containing compounds, including Bortezomib (Velcade), the first boronic acid-based drug for treating multiple myeloma, as well as Tavaborate, Ixazomib, Crisaborole, and Vaborbactam [16]. Boron compounds have shown promising therapeutic potential in cancers such as breast, glioblastoma, lung, prostate, and liver cancers [17,18,19,20]. Sodium pentaborate (NaB; NaB_5_O_8_), a boron-based compound, has demonstrated efficacy in various biological contexts, including obesity management, wound healing, and tumor suppression [21,22,23].

Previous studies demonstrated that CBD, CBG, and sodium pentaborate individually induce concentration-dependent reductions in cell viability in HCT-116 and HT-29 colorectal cancer cell lines and modulate apoptosis- and proliferation-associated responses [13,23]. In particular, cannabinoids were reported to exert anti-cancer activity through regulation of oxidative stress and cell death pathways, whereas sodium pentaborate exhibited anti-proliferative effects in colorectal cancer models. These findings provided the rationale for selecting HCT-116 and HT-29 cells and evaluating the combined effects of NaB_5_O_8_ with CBD and CBG in the present study. Building upon this, the present study aimed to investigate the combined therapeutic effects of NaB with cannabinoid derivatives (CBD and CBG) in these cell lines. Our results demonstrated that the combination of NaB with CBD elicited a synergistic reduction in cell viability and significantly increased early apoptosis in both HCT-116 and HT-29 cell lines. Conversely, the combination of NaB with CBG exhibited antagonistic effects, showing no synergistic potential in either cell line. These findings highlight the importance of compound selection in combinatory cancer therapy and support further exploration of NaB and CBD as a potential adjunct treatment strategy in colorectal cancer.

## 2. Results

### 2.1. Combination Treatment Analysis: NaB and CBD/CBG in HCT-116 and HT-29 Cancer Cell Lines

As demonstrated in our previous studies, both HCT-116 and HT-29 cell lines are responsive to cannabidiol (CBD), cannabigerol (CBG), and sodium pentaborate (NaB), each of which significantly affects cell viability. The experimentally determined IC_50_ values of the individual compounds are presented in Table 1. Here, we evaluated the combined effects of NaB with CBD or CBG on colorectal cancer cell growth.

**Figure 2:**
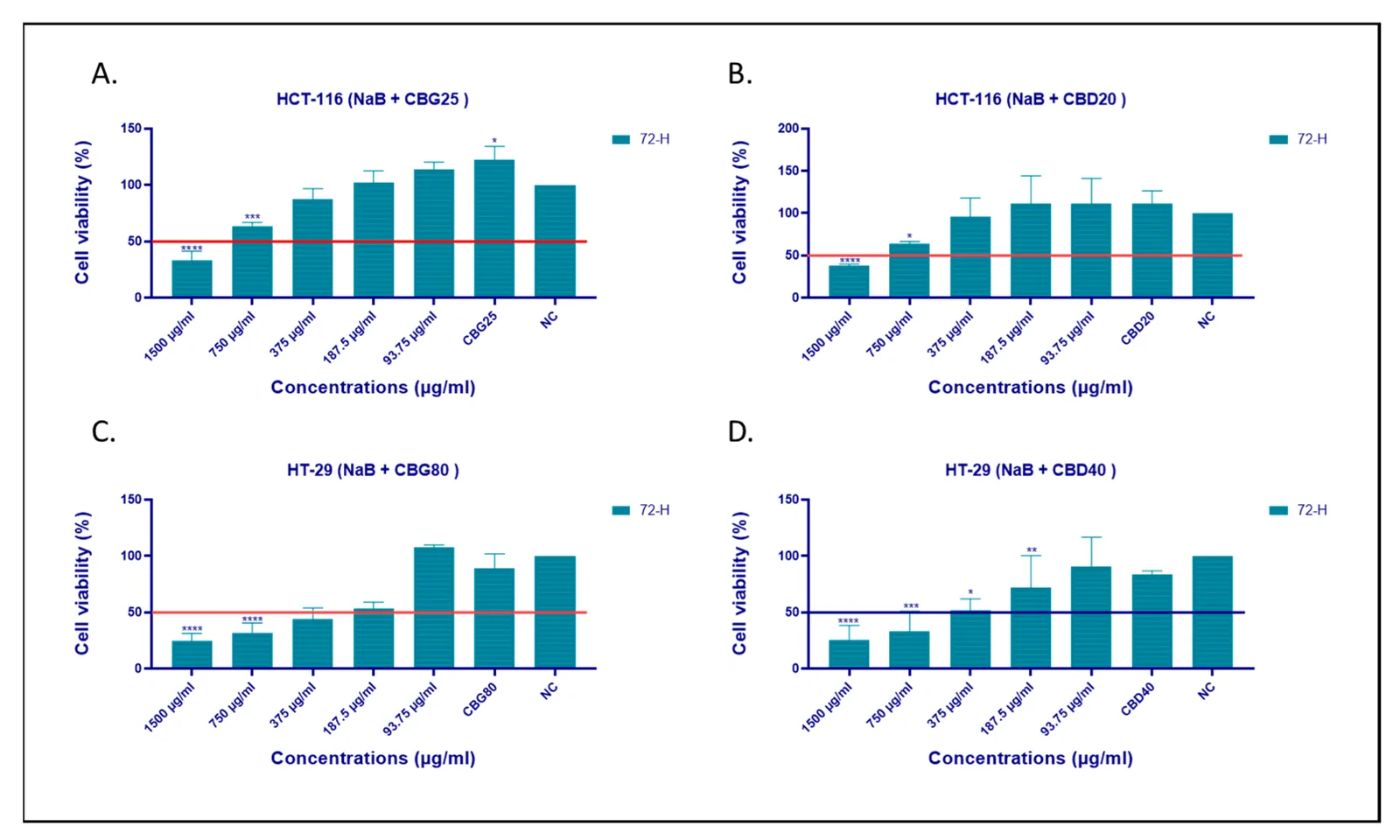
Cell viability (%) of HCT-116 and HT-29 cells after 72 h treatment with NaB combined with CBG or CBD: (**A**) NaB + CBG in HCT-116, (**B**) NaB + CBD in HCT-116, (**C**) NaB + CBG in HT-29, and (**D**) NaB + CBD in HT-29. Viability was determined by MTS assay and normalized to untreated controls. Data are shown as mean ± SD (*n* = 3). The red line represents 50% viability. * *p* < 0.05; ** *p* < 0.01; *** *p* < 0.001; **** *p* < 0.0001.

As illustrated in Figure 2, cells were initially treated for 72 h with a range of NaB concentrations together with fixed non-cytotoxic concentrations of CBD or CBG. Following the reviewer’s recommendation, the combination experiments were repeated using a fixed-ratio Chou–Talalay design based on the experimentally determined IC_50_ values of the individual compounds. The resulting dose–response curves and combination index analyses are presented in Supplementary Figure S1.

The combinational response was strongly cell line dependent. In HCT-116 cells, the fixed-ratio combination analysis predominantly demonstrated additive or antagonistic interactions, although synergistic interactions were observed at selected dose combinations. In HT-29 cells, NaB–CBD and NaB–CBG combinations produced enhanced antiproliferative effects compared with NaB alone. Combination index analysis demonstrated dose-dependent interactions, with synergistic effects observed only at selected dose combinations, whereas additive or antagonistic interactions were detected at other concentration levels.

These findings indicate that the response to NaB–cannabinoid co-treatment is highly dependent on both cellular context and drug concentration ratio, highlighting the importance of dose optimization for combination therapy.

### 2.2. Combination Apoptosis Analysis: NaB and CBD/CBG in HCT-116 and HT-29 Cancer Cell Lines

**Figure 3:**
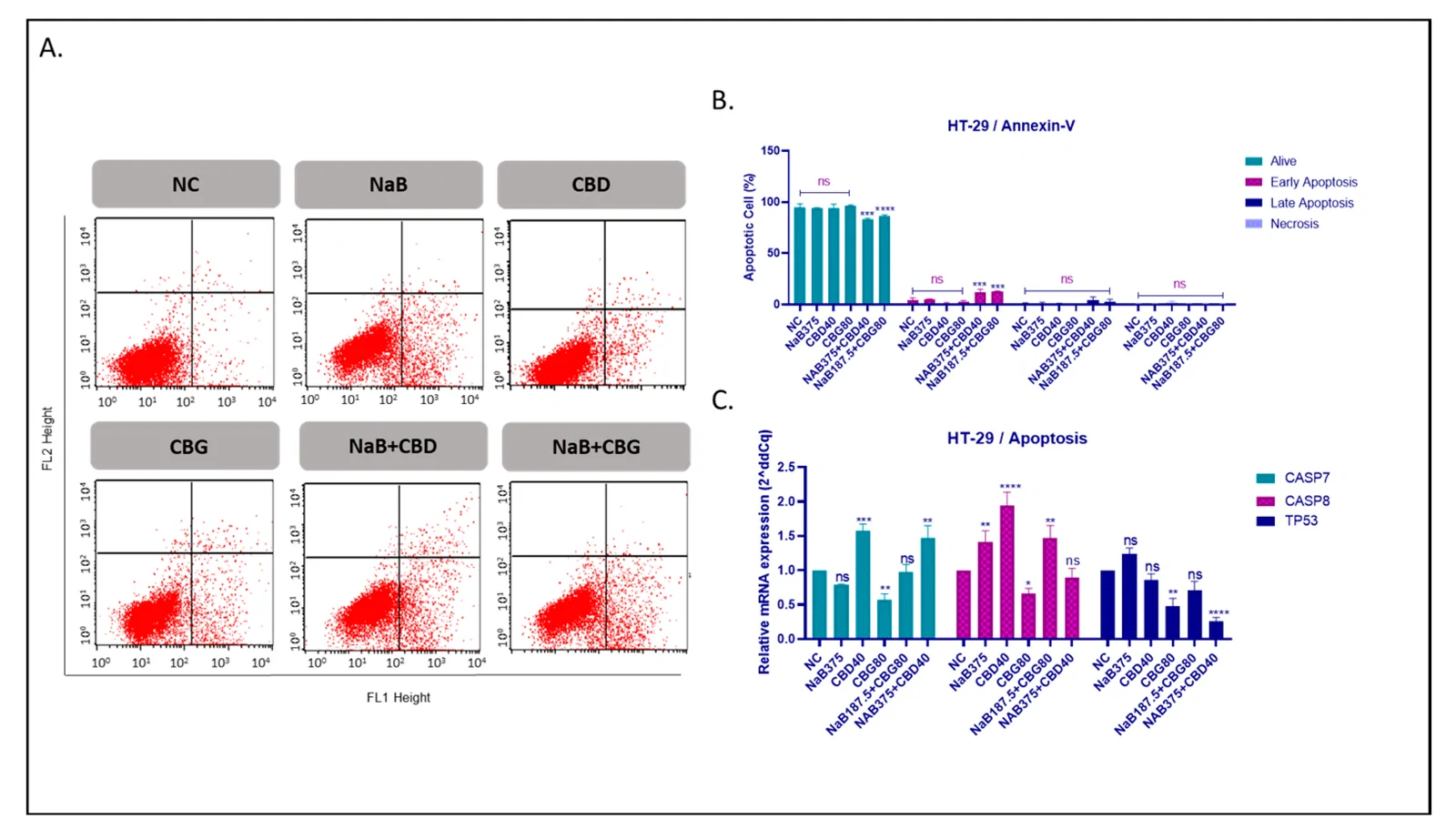
Flow cytometric analysis of apoptosis in HT-29 cells following treatment with NaB, CBD, CBG, and their combinations for 72 h using an Annexin V-FITC/PI staining assay. (**A**) Representative dot plots showing Annexin V/PI staining profiles for negative control (NC), NaB, CBD, CBG, NaB + CBD, and NaB + CBG treatment groups. (**B**) Quantitative analysis of apoptotic cell populations, including viable, early apoptotic, late apoptotic, and necrotic cells, expressed as percentages. (**C**) Relative mRNA expression levels of apoptosis-related genes (CASP7, CASP8, and TP53) in HT-29 cells determined by qPCR and normalized to the NC group. Data are presented as mean ± SD (n = 3). ns, not significant; * *p* < 0.05; ** *p* < 0.01; *** *p* < 0.001; **** *p* < 0.0001 vs. NC.

**Figure 4:**
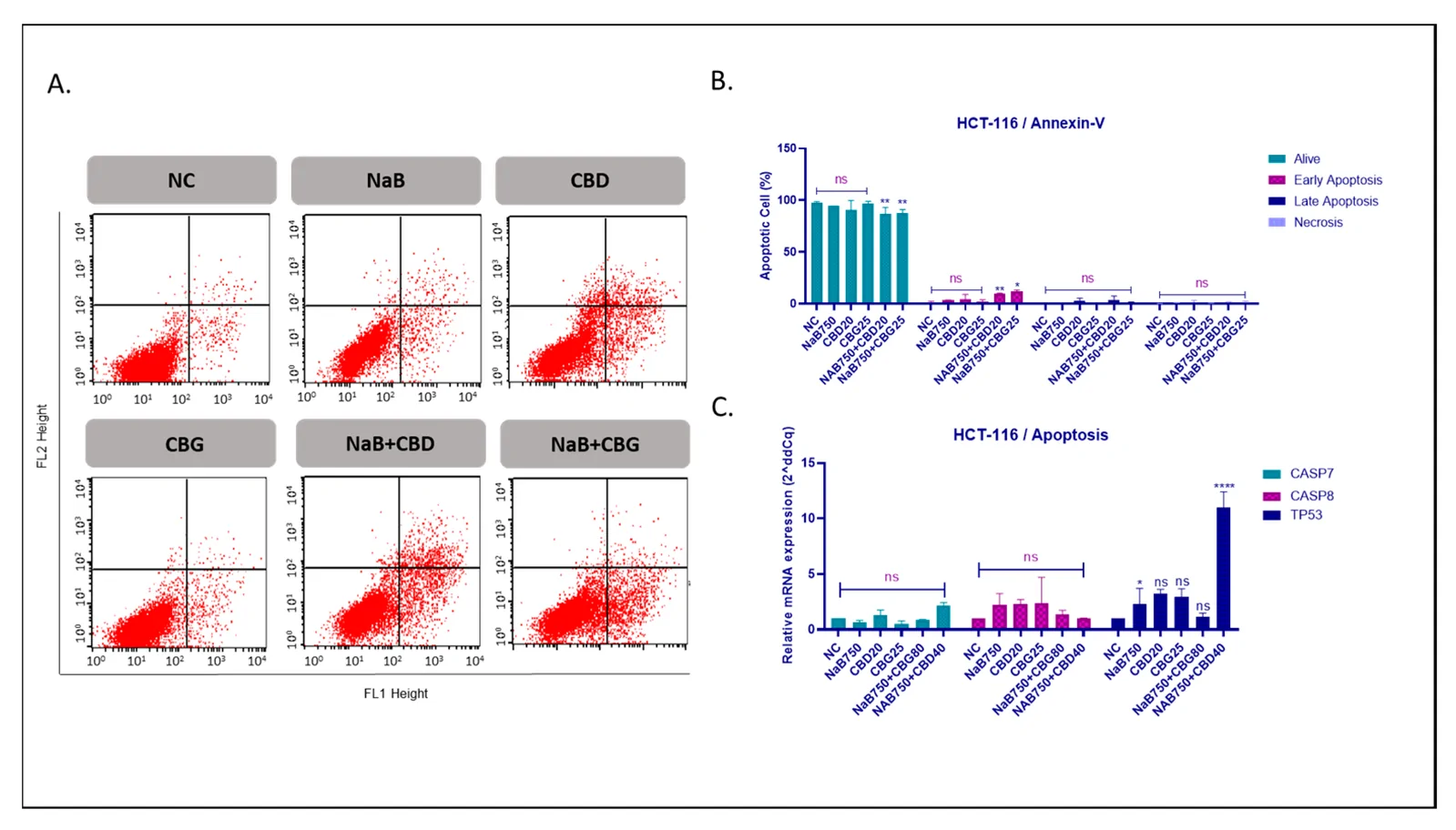
Flow cytometric analysis of apoptosis in HCT-116 cells following treatment with NaB, CBD, CBG, and their combinations for 72 h using an Annexin V-FITC/PI staining assay. (**A**) Representative dot plots showing Annexin V/PI staining profiles for negative control (NC), NaB, CBD, CBG, NaB + CBD, and NaB + CBG treatment groups. (**B**) Quantitative analysis of apoptotic cell populations, including viable, early apoptotic, late apoptotic, and necrotic cells, expressed as percentages. (**C**) Relative mRNA expression levels of apoptosis-related genes (CASP7, CASP8, and TP53) in HCT-116 cells determined by qPCR and normalized to the NC group. Data are presented as mean ± SD (n = 3). ns, not significant; * *p* < 0.05; ** *p* < 0.01; **** *p* < 0.0001 vs. NC.

**Figure 5:**
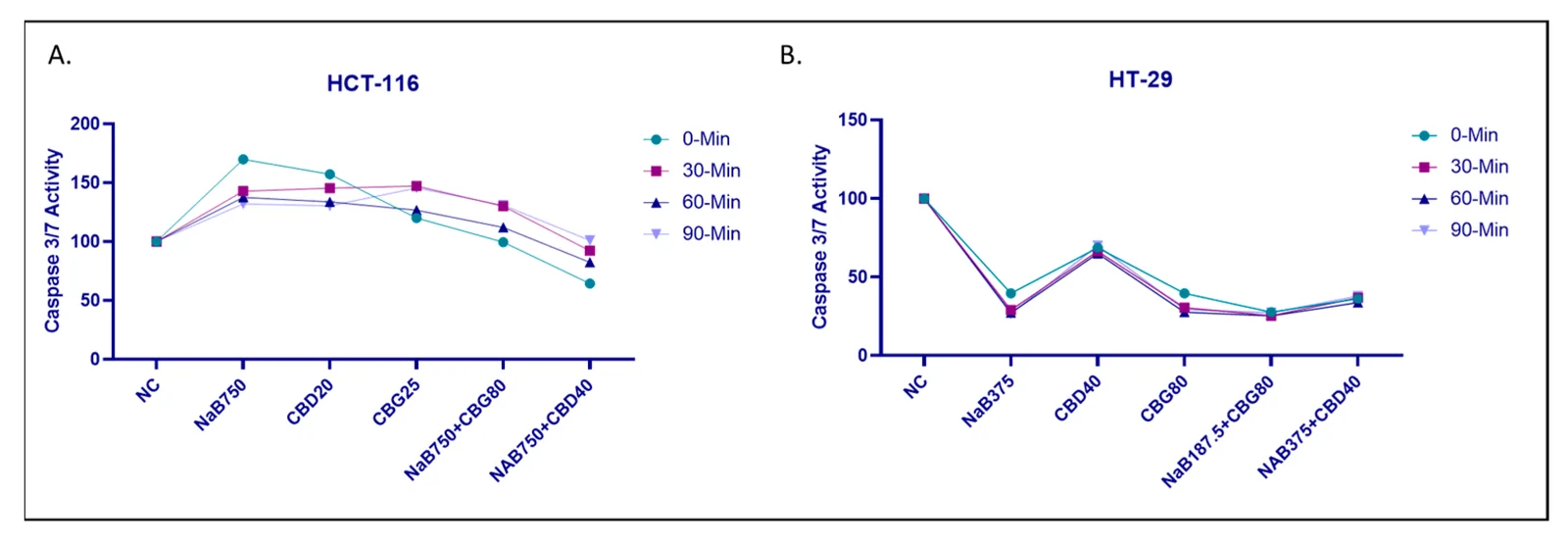
Caspase-3/7 activity in HCT-116 and HT-29 cells after treatment with NaB, CBD, CBG, and their combinations. Activity was measured at 0, 30, 60, and 90 min and normalized to the negative control (NC). (**A**) HCT-116 cell line; (**B**) HT-29 cell line.

The effects of NaB, CBD, CBG, and their combinations on apoptosis were evaluated in HCT-116 and HT-29 colorectal cancer cell lines using Annexin V/PI staining, apoptosis-related gene expression analysis, and caspase-3/7 activity assays (Figure 3, Figure 4 and Figure 5). Annexin V/PI analysis showed that combination treatments increased the proportion of early apoptotic cells in both cell lines, with a more pronounced effect in HT-29 cells. In HT-29 cells, early apoptosis increased to 12.49% following NaB + CBG and to 12.14% following NaB + CBD treatment (*p* < 0.01). In HCT-116 cells, early apoptosis increased to 9.80% with NaB + CBD and to 11.80% with NaB + CBG. Late apoptosis and necrosis were not markedly altered in either cell line.

qPCR analysis revealed cell line-dependent modulation of apoptosis-related genes. In HT-29 cells, NaB + CBG increased CASP7 expression (~1.47-fold; *p* < 0.01), whereas changes in CASP8 were limited. In HCT-116 cells, NaB + CBG markedly increased TP53 expression (~11.02-fold; *p* < 0.001), while CASP7 and CASP8 exhibited comparatively modest changes.

Consistent with these findings, caspase-3/7 activity increased over time in HCT-116 cells following NaB, CBD, and CBG monotherapies, whereas combination treatments produced a more moderate caspase-3/7 response relative to single agents. In HT-29 cells, caspase-3/7 activity did not show a marked increase under the tested conditions.

### 2.3. Combination Cell Cycle Analysis: NaB and CBD/CBG in HCT-116 and HT-29 Cancer Cell Lines

To determine whether the observed apoptotic responses were associated with changes in cell cycle progression, flow cytometric cell cycle analysis and qPCR assessment of proliferation-related markers were conducted in both HCT-116 and HT-29 cell lines.

**Figure 6:**
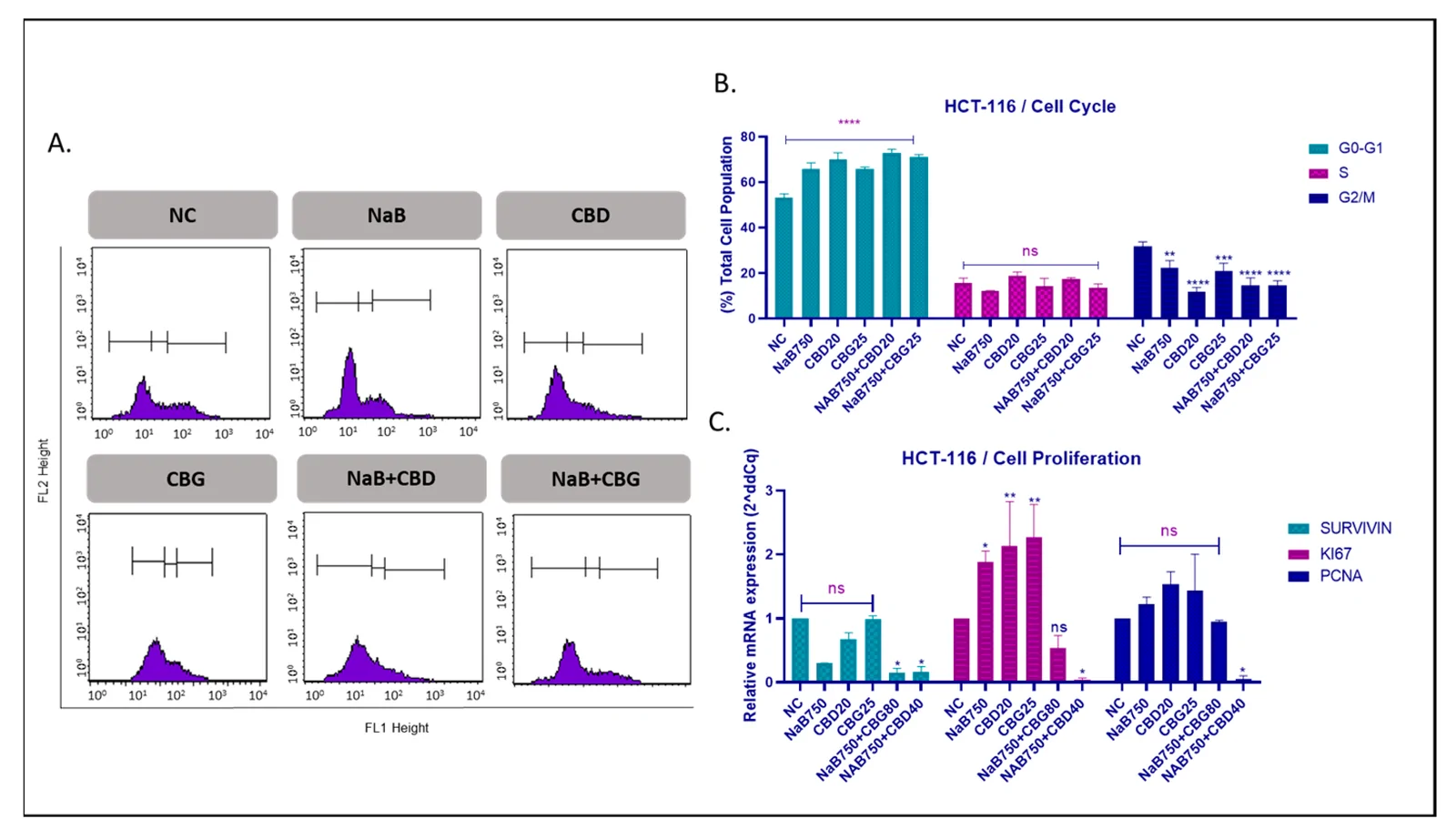
Effects of NaB, CBD, CBG, and their combinations on cell cycle distribution and proliferation-related gene expression in HCT-116 cells. (**A**) Representative DNA content histograms showing cell cycle profiles for negative control (NC), NaB, CBD, CBG, NaB + CBD, and NaB + CBG treatment groups after 72 h. (**B**) Quantitative analysis of cell cycle phase distribution (G0/G1, S, and G2/M) expressed as percentages. (**C**) Relative mRNA expression levels of proliferation-associated genes (SURVIVIN, KI67, and PCNA). Data are presented as mean ± SD (n = 3). ns, not significant; * *p* < 0.05; ** *p* < 0.01; *** *p* < 0.001; **** *p* < 0.0001.

In HCT-116 cells, combination treatments—most notably NaB + CBG—induced a clear redistribution of cells across the cell cycle phases (Figure 6). Flow cytometry revealed a significant accumulation of cells in the G0/G1 phase accompanied by a marked reduction in the G2/M fraction, indicating a pronounced cell cycle arrest profile. In parallel, qPCR analysis demonstrated a coordinated antiproliferative transcriptional response in the NaB + CBG group, with significant downregulation of SURVIVIN, KI67, and PCNA expression levels. These results suggest that the NaB + CBG combination exerts a robust growth-suppressive effect in HCT-116 cells.

**Figure 7:**
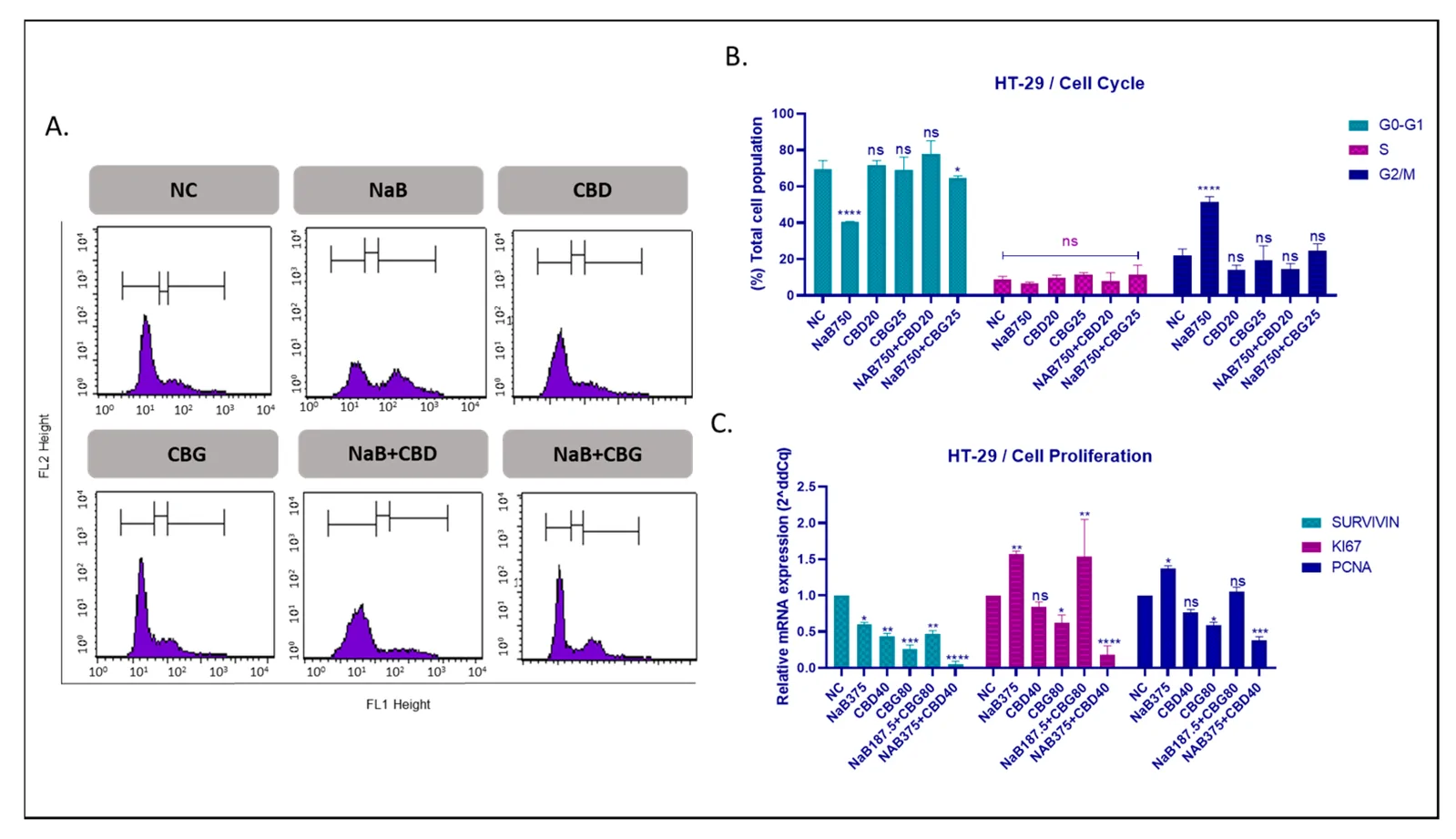
Effects of NaB, CBD, CBG, and their combinations on cell cycle distribution and proliferation-related gene expression in HT-29 cells. (**A**) Representative DNA content histograms showing cell cycle profiles for negative control (NC), NaB, CBD, CBG, NaB + CBD, and NaB + CBG treatment groups after 72 h. (**B**) Quantitative analysis of cell cycle phase distribution (G0/G1, S, and G2/M) expressed as percentages. (**C**) Relative mRNA expression levels of proliferation-associated genes (SURVIVIN, KI67, and PCNA). Data are presented as mean ± SD (n = 3). ns, not significant; * *p* < 0.05; ** *p* < 0.01; *** *p* < 0.001; **** *p* < 0.0001.

In HT-29 cells, by contrast, flow cytometric analysis did not reveal a distinct phase-specific arrest pattern across any treatment group, and overall cell cycle distribution remained largely comparable to the negative control (Figure 7). Nevertheless, qPCR analysis indicated treatment-responsive modulation of proliferation-associated genes at the transcriptional level. Specifically, NaB + CBG significantly reduced SURVIVIN expression, whereas NaB + CBD significantly increased KI67 levels; PCNA expression did not exhibit statistically significant changes among the treatment conditions. Although these transcriptional alterations reflect regulation of proliferation-related pathways in HT-29 cells, they did not translate into a corresponding redistribution of cells across cell cycle phases. Accordingly, in HT-29 cells, antiproliferative responses appear to be more limited.

### 2.4. Combination Ferroptosis Analysis: NaB and CBD/CBG in HCT-116 and HT-29 Cancer Cell Lines

The effects of NaB, CBD, CBG, and their combinations on ferroptosis-associated molecular pathways were evaluated in HCT-116 and HT-29 cells by qPCR analysis of SLC11A2, SLC3A2, SLC7A11, KEAP1, HMOX4, and NRF2 gene expression.

**Figure 8:**
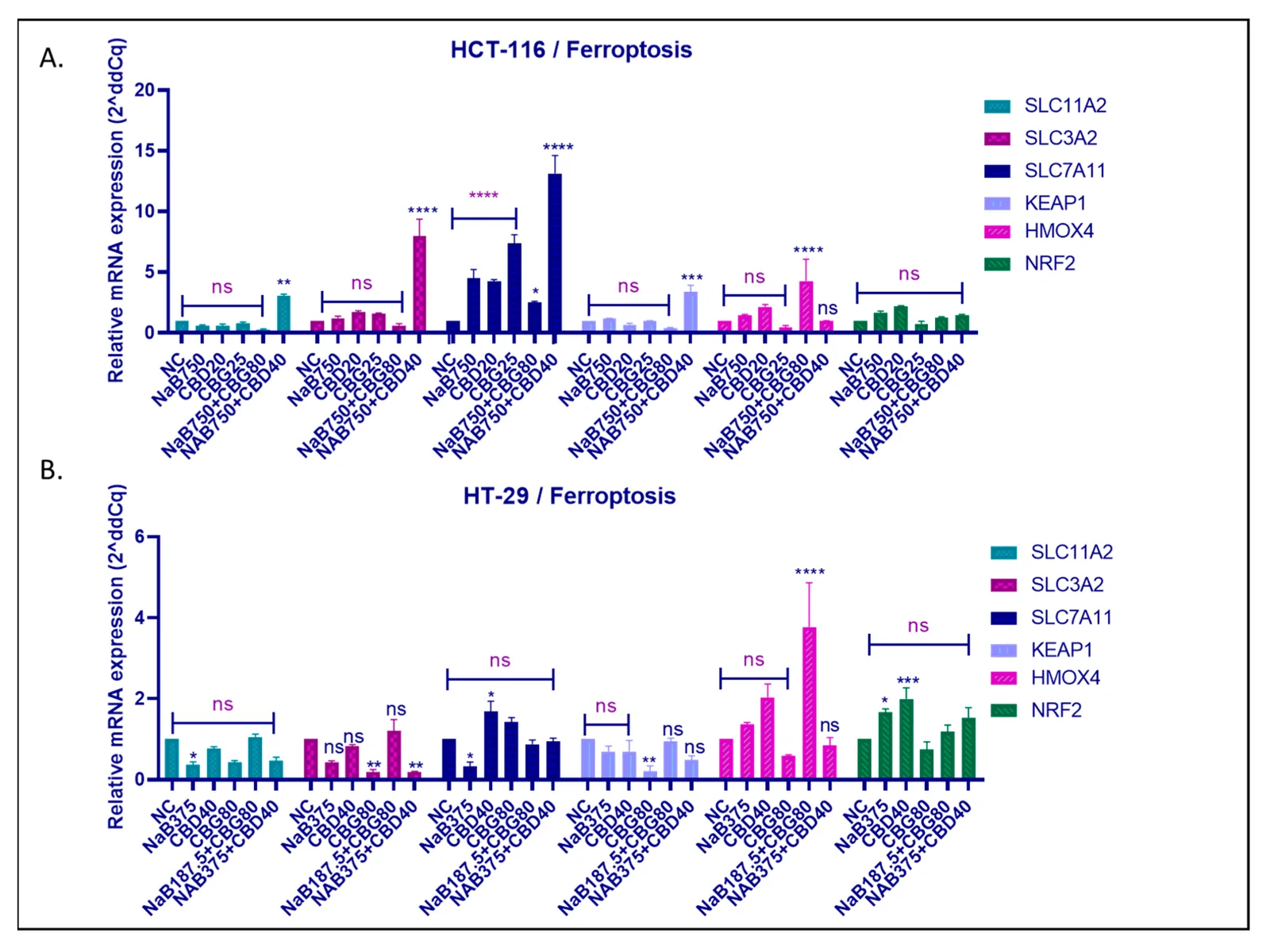
(**A**) Ferroptosis-related gene expression in HCT-116 cells after treatment with NaB, CBD, CBG, and their combinations for 72 h.; (**B**) Ferroptosis-related gene expression in HT-29 cells after treatment with NaB, CBD, CBG, and their combinations for 72 h. Relative mRNA levels of SLC11A2, SLC3A2, SLC7A11, KEAP1, HMOX4, and NRF2 were analyzed by qPCR. Data are mean ± SD (n = 3). * *p* < 0.05, ** *p* < 0.01, *** *p* < 0.001, **** *p* < 0.0001 vs. NC.

In HCT-116 cells, NaB-based combination treatments induced distinct transcriptional alterations in ferroptosis-associated genes (Figure 8A). The NaB + CBG combination produced the most prominent response, characterized by significant upregulation of SLC7A11 (≈13.14-fold), SLC3A2 (≈7.98-fold), and SLC11A2 (≈3.01-fold), together with increased KEAP1 expression (≈3.38-fold). In contrast, the NaB + CBD combination predominantly increased SLC7A11 (≈2.48-fold) and HMOX4 (≈4.27-fold) expression, whereas the remaining ferroptosis-associated genes showed comparatively modest changes.

In HT-29 cells, the transcriptional response was more limited. The NaB + CBD treatment significantly decreased SLC3A2 expression (≈0.19-fold), whereas the NaB + CBG combination significantly increased HMOX4 expression (~3.77-fold) (Figure 8B).

Taken together, NaB–CBD and particularly NaB–CBG combinations induced gene-selective and cell line-dependent alterations in ferroptosis-associated gene expression. These transcriptional changes indicate modulation of pathways involved in redox homeostasis and iron metabolism, suggesting a differential ferroptosis-related molecular response between the two cell lines.

## 3. Discussion

In the present study, we investigated the combined anti-cancer effects of sodium pentaborate (NaB) with the phytocannabinoids cannabidiol (CBD) and cannabigerol (CBG) in two human colorectal cancer cell lines, HCT-116 and HT-29, representing distinct molecular and phenotypic backgrounds. Our findings demonstrate that NaB–cannabinoid combinations enhance anti-proliferative and pro-apoptotic responses compared with single-agent treatments, although the magnitude and nature of these effects were strongly cell line dependent. Notably, the interaction between NaB and cannabinoids was highly dose-dependent. HT-29 cells exhibited enhanced treatment responses at selected combination dose levels, whereas only limited combinational benefit was observed in HCT-116 cells.

The divergent combinational responses observed between HCT-116 and HT-29 cells are likely attributable to their distinct molecular backgrounds. Although both lines originate from colorectal tumors, they represent biologically different models. HCT-116 cells are commonly described as a microsatellite instability–high (MSI-high) colorectal cancer model with functional wild-type p53, whereas HT-29 cells are microsatellite stable and harbor oncogenic alterations including the BRAFV600E mutation together with TP53 dysfunction [24].

Notably, mutant p53 has been widely associated with genomic instability and deregulated cell cycle control, as gain-of-function TP53 variants can promote chromosomal instability and increased susceptibility to cellular stress in cancer cells [25]. Such defects may lower the apoptotic threshold and enhance vulnerability to combinational treatments. Consistent with this interpretation NaB–cannabinoid co-treatment produced enhanced antiproliferative responses at selected combination dose levels in HT-29 cells, whereas only limited benefit was observed in HCT-116 cells, indicating that the therapeutic efficacy of these combinations may depend strongly on tumor-specific molecular context rather than representing a universal response.

Our analyses further support this interpretation. In HT-29 cells, NaB–cannabinoid co-treatment markedly increased early apoptotic populations and produced stronger growth inhibition compared with monotherapies, indicating enhanced biological activity compared with monotherapies. Interestingly, although early apoptotic populations increased following combination treatment, this effect was not uniformly accompanied by elevated caspase-3/7 activity. This discrepancy may reflect differences in the temporal dynamics of apoptotic progression or indicate that early apoptotic responses do not necessarily require proportional activation of downstream caspase signaling under all experimental conditions. Therefore, the observed findings may represent heterogeneous or partially caspase-independent apoptotic responses. These observations are consistent with previous reports demonstrating that phytocannabinoids, particularly CBD and CBG, exert anti-cancer effects through induction of apoptosis, oxidative stress, and suppression of proliferative signaling across multiple cancer models [13,26,27]. Cannabinoids have been shown to disrupt mitochondrial homeostasis, elevate reactive oxygen species levels, and activate caspase-dependent cell death pathways, thereby sensitizing tumor cells to additional therapeutic stress. In parallel, boron-containing compounds have been reported to exhibit anti-proliferative and metabolic regulatory properties, interfering with tumor cell growth and survival and enhancing stress-mediated cytotoxicity [28,29]. The simultaneous targeting of complementary survival mechanisms by NaB and cannabinoids may therefore amplify apoptotic signaling and reduce cellular adaptability, contributing to the dose-dependent biological responses observed in HT-29 cells.

In contrast, HCT-116 cells displayed only modest alterations in apoptotic fractions and minimal changes in cell cycle distribution following combination treatment, consistent with the predominantly additive or antagonistic interaction profile identified by the fixed-ratio combination analysis. This limited response suggests that intrinsic resistance mechanisms or alternative survival pathways may attenuate combinational efficacy in this genetic background. Moreover, the transcriptional modulation of ferroptosis-related genes observed predominantly in HT-29 cells indicates that additional redox- and iron-dependent stress responses may contribute to treatment sensitivity, as ferroptosis is increasingly recognized as a critical vulnerability in cancer cells [30].

Beyond its mechanistic relevance, ferroptosis has emerged as a promising therapeutic strategy in colorectal cancer because it may provide an alternative vulnerability in tumors exhibiting resistance to apoptosis or conventional treatment approaches. Recent studies suggest that induction of ferroptosis can suppress tumor progression by disrupting redox homeostasis, promoting iron-dependent lipid peroxidation, and overcoming adaptive survival mechanisms in CRC cells. In addition, ferroptosis-associated pathways have recently been recognized as promising therapeutic targets for improving treatment sensitivity and overcoming drug resistance in colorectal cancer [31,32]. In this context, modulation of ferroptosis-associated genes may be biologically relevant and may reflect alterations in cellular stress adaptation and redox regulation. However, functional validation will be required to determine whether ferroptotic cell death directly contributes to the observed treatment responses [33,34].

The observed transcriptional alterations across apoptosis-, proliferation-, and ferroptosis-associated markers may collectively suggest coordinated anti-tumor responses induced by combination treatment. However, these findings should be interpreted cautiously, as mRNA expression does not necessarily reflect protein abundance or functional pathway activation, particularly in pathways regulated at post-translational levels. Therefore, the present results should be considered as transcriptional evidence associated with pathway modulation rather than definitive mechanistic confirmation. Future studies integrating protein-level validation (e.g., Western blotting for cleaved caspase-3, GPX4, and NRF2) together with complementary functional assays, including lipid peroxidation measurements, iron dependency analyses, and rescue experiments using ferroptosis inhibitors, will be necessary to further validate the underlying molecular mechanisms. Together, these findings provide an initial framework for understanding the context-dependent responses induced by NaB–cannabinoid combination treatment in colorectal cancer models. Although HCT-116 and HT-29 represent distinct molecular backgrounds of colorectal cancer, the use of only two cell lines limits broader generalization across the heterogeneous landscape of CRC. Another limitation of the present study is the absence of a non-cancerous colorectal cell model. Future studies incorporating normal colorectal epithelial cells will be important to evaluate treatment selectivity and further assess the translational relevance of NaB–cannabinoid combination treatment. Future studies incorporating additional colorectal cancer models, including three-dimensional cultures and in vivo systems, will be important to further validate the context-dependent responses observed in this study.

## 4. Materials and Methods

### 4.1. Cell Lines and Cell Culture Conditions

HT-29 (HTB-38, human colorectal adenocarcinoma cell lines) and HCT-116 (CRL-247, human colorectal adenocarcinoma cell lines) were originally purchased from American Type Culture Collection (ATCC, Rockville, MD, USA). HT-29 cell lines were cultured in Roswell Park Memorial Institute medium (RPMI, #11875093, Invitrogen, Gibco, Paisley, UK). HCT-116 cell lines were cultured in Dulbecco’s Modified Eagle’s Medium (DMEM, #41966-029, Invitrogen, Gibco, UK). Each medium was supplemented with %1 Penicillin/Streptomycin/Amphotericin (PSA, Invitrogen, Gibco, UK) and %10 fetal bovine serum (FBS, #10500-064, Invitrogen, Gibco, UK). Cells were maintained at 37 °C and 5% CO_2_ in a humidified incubator.

### 4.2. Cytotoxicity Assay

Cells were seeded into 96-well plates at a density of 5 × 10^3^ cells/well and allowed to attach overnight. Based on previously published single-agent cytotoxicity profiles of NaB, CBD, and CBG from our earlier studies [13,23], cannabinoids were applied at fixed, non-cytotoxic concentrations, while CBG was tested at increasing concentrations (3000–93.75 µg/mL). Based on our previously published single-agent dose–response experiments, cannabinoid concentrations maintaining >80% cell viability after 72 h treatment were defined as non-cytotoxic and selected for combination experiments. Cell line-specific concentrations were used to account for differential sensitivity to cannabinoids. Cells were incubated for 72 h under standard culture conditions.

Cell viability was assessed using the CellTiter 96^®^ AQueous One Solution MTS assay (Promega, Southampton, UK) according to the manufacturer’s instructions. Treatment media were replaced with fresh medium containing MTS reagent and incubated for 90 min at 37 °C. Absorbance was measured at 490 nm using a microplate reader (BioTek, Winooski, VT, USA), and viability values were normalized to untreated controls. IC_50_ values were calculated using GraphPad Prism software 8.0.1.

### 4.3. Combination Index Analysis

For the additional fixed-ratio experiments, combination effects were quantitatively evaluated according to the median-effect principle of Chou and Talalay using CompuSyn software (version 1.0). Fraction affected (Fa) values obtained from the MTS assay were used to calculate the combination index (CI). CI values were interpreted as follows: CI < 1, synergism; CI = 1, additive effect; and CI > 1, antagonism.

### 4.4. Annexin V Assay

Apoptosis was evaluated using an Annexin V-FITC/propidium iodide (PI) staining assay. HCT-116 and HT-29 cells were seeded into T25 flasks at a density of 2.5 × 10^5^ cells per flask and allowed to attach overnight. Cells were then treated with the corresponding combination of IC_50_ concentrations for 72 h under standard culture conditions. Following treatment, cells were harvested, washed twice with ice-cold PBS, and re-suspended in Annexin V binding buffer. Cells were first incubated with Annexin V-FITC for 15 min at room temperature in the dark. Propidium iodide was subsequently added immediately before flow cytometric acquisition. Apoptotic populations were quantified using a FACSCalibur flow cytometer (BD Biosciences, Franklin Lakes, NJ, USA), and the percentages of viable, early apoptotic, late apoptotic, and necrotic cells were determined.

### 4.5. Cell Cycle Analysis

Cell cycle distribution was analyzed by flow cytometry. HCT-116 and HT-29 cells were seeded into T25 flasks at a density of 2.5 × 10^5^ cells per flask and allowed to attach overnight. Following treatment for 72 h, cells were harvested, washed with PBS, and fixed in 70% ice-cold ethanol for at least 2 h at −20 °C. Fixed cells were permeabilized with 0.1% Triton X-100 and incubated with RNase A (20 µg/mL) at room temperature for 30 min. Cells were then stained with propidium iodide (PI) and analyzed immediately by flow cytometry using a FACSCalibur system (BD Biosciences, San Jose, CA, USA). DNA content histograms were used to determine the percentages of cells in G0/G1, S, and G2/M phases.

### 4.6. Caspase-3/7 Activity

Caspase-3/7 activity was measured using the Caspase-Glo^®^ 3/7 assay system (Promega, Madison, WI, USA) according to the manufacturer’s instructions. HCT-116 and HT-29 cells were seeded into white 96-well plates and allowed to attach overnight. Cells were then treated with the corresponding combination IC_50_ concentrations for 72 h. Following treatment, Caspase-Glo^®^ reagent was added directly to each well and incubated for the recommended time at room temperature. Luminescence was measured using a microplate luminometer (Varioskan Lux, Thermo Scientific, Waltham, MA, USA). Caspase activity was expressed relative to untreated controls.

### 4.7. Real-Time PCR

Total RNA was isolated using an RNA extraction kit (Macherey-Nagel, Düren, Germany) according to the manufacturer’s instructions. cDNA synthesis was performed from total RNA using the QuantiTect Reverse Transcription Kit (Qiagen, Hilden, Germany).RT-PCR was performed using SYBR Green (#4309155, Thermo Fisher, Waltham, MA, USA) and assayed in triplicate using the iCycler RT-PCR detection system (Bio-Rad, Hercules, CA, USA). The expression levels were normalized with respect to RPL30 (Ribosomal Protein L30) gene (F: 5′-ACAGCATGCGGAAAATACTAC-3′ R: 5′-AAAGGAAAATTTTGCAGGTTT-3′) levels. Genes and their corresponding primer sequences used in this study as follows; Tumor protein53 (TP53) (F: 5′-GCCCAACAACACCAGCTCCT-3′ R: 5′-CCTGGGCATCCTTGAGTTCC-3′), Caspase-7 (F: 5′-TCAGTGGATGCTAAGCCAGACC-3′ R: 5′-CGAACGCCCATACCTGTCAC-3′), Caspase-8 (F: 5′-GCCACCCGGCTTCAGAATGGC-3′ R: 5′-TATGGGCCATCTGCTGTTGGCAGT-3′), Baculoviral inhibitor of apoptosis repeat-containing 5 (BIRC5 or Survivin) (F: 5′-TCTTCACCGCTTTGCTTTC-3′ R: 5′-CGCACTTTCTCCGCAGTTTC-3′), Marker of Proliferation Kiel 67 (Ki-67) (F: 5′-GAAAGAGTGGCAACCTGCCTTC-3′ R: 5′-GCACCAAGTTTTACTACATCTGCC-3′), PCNA (F: 5′-CAAGTAATGTCGATAAAGAGGAGG-3′ R: 5′-GTGTCACCGTTGAAGAGAGTGG-3′), HMOX1 (F: 5′-CCAGGCAGAGAATGCTGAGTTC-3′ R: 5′-AA-GACTGGGCTCTCCTTGTTGC-3′), SLC7A11 (F: 5′-TCATTGGAGCAGGAATCTTCA-3′ R: 5′-TTCAGCATAAGACAAAGCTCCA-3′), SLC3A2 (F: 5′-CTGGTGCCGTGGTCATAATC-3′ R: 5′-GCTCAGGTAATCGAGACGCC-3′), SLC11A2 (F: 5′-TCCATTCCTGAGGAGGAG-TA-3′ R: 5′-CAGACTGCAAATCGGATTCA-3′), KEAP1 (F: 5′-AG-GTATGAGCCAGAGCGGGATG-3′ R: 5′-AGGTATGAGCCAGAGCGGGATG-3′), NRF2 (F: 5′-CCAGCACATCCAGTCAGAA-3′ R: 5′-CGTAGCCGAAGAAACCTCA-3′ ). The fold changes for each sample were determined using the 2 [−Delta C(T)] method.

## 5. Conclusions

In conclusion, our findings indicate that co-treatment with NaB and phytocannabinoids provides a cell line-specific therapeutic strategy in colorectal cancer. The biological responses to NaB–cannabinoid combinations were highly dependent on both cellular context and drug concentration ratio. Although synergistic interactions were observed at selected dose combinations, the overall response varied across the tested concentration range. The concurrent modulation of apoptosis-, proliferation-, and ferroptosis-associated pathways suggests that this combination may offer therapeutic potential in tumors with susceptible molecular backgrounds. Validation of these findings in more physiologically relevant three-dimensional tumor models and in vivo systems will be important to further assess the translational potential of this approach.

## Figures and Tables

**Table 1:** IC50 values of NaB alone and in combination treatments in colorectal HCT-116 and HT-29 cancer cell lines.

Compounds	HCT-116 IC50	HT-29 IC50
NaB	3388 μM (1000 μg/mL) [23]	1694 μM (500 μg/mL) [23]
CBD	159.2 μM [13]	143.3 μM [13]
CBG	94.79 μM [13]	284.37 μM [13]
NaB + CBD	2280 μM (672.7 μg/mL)	945 μM (279.0 μg/mL)
NaB + CBG	2547 μM (751.5 μg/mL)	605 μM (178.5 μg/mL)

## Data Availability

Data is contained in the paper.

## Supplementary Materials

The following supporting information can be downloaded at: https://www.mdpi.com/article/10.3390/molecules31183206/s1, Figure S1: Fixed-ratio combination analysis of NaB with CBD or CBG in HCT-116 and HT-29 cells. (A–D) Cell viability after 72 h treatment. Data are shown as mean ± SD (n = 3). The red line represents 50% viability. (E–H) CompuSyn dose–effect curves. (I–L) Median-effect plots generated according to the Chou–Talalay method.

## References

[1] Fleming M., Ravula S., Tatishchev S.F., Wang H.L. (2012). Colorectal carcinoma: Pathologic aspects. J. Gastrointest. Oncol..

[2] Mph R.L.S., Sandeep N., Mbbs W., Cercek A., Smith R.A. (2023). Colorectal cancer statistics. CA Cancer J. Clin..

[3] Molinari C., Marisi G., Passardi A., Matteucci L., De Maio G., Ulivi P. (2018). Heterogeneity in Colorectal Cancer: A Challenge for Personalized Medicine?. Int. J. Mol. Sci..

[4] Zhou H., Liu Z., Wang Y., Wen X., Amador E.H., Yuan L., Ran X., Xiong L., Ran Y. (2022). Colorectal liver metastasis: Molecular mechanism and interventional therapy. Signal Transduct. Target. Ther..

[5] Kleckner A.S., Kleckner I.R., Kamen C.S., Tejani M.A., Janelsins M.C., Morrow G.R., Peppone L.J. (2019). Opportunities for cannabis in supportive care in cancer. Ther. Adv. Med. Oncol..

[6] Kis B., Ifrim F.C., Buda V., Avram S., Pavel I.Z., Antal D., Paunescu V., Dehelean C.A., Ardelean F., Diaconeasa Z. (2019). Cannabidiol—From Plant to Human Body: A Promising Bioactive Molecule with Multi-Target Effects in Cancer. Int. J. Mol. Sci..

[7] Manuscript A., Cells C., Phosphatase I. (2012). Induction of Apoptosis by Cannabinoids in Prostate and Colon Cancer Cells Is Phosphatase Dependent. Anticancer Res..

[8] Mcallister S.D., Soroceanu L., Desprez P. (2015). The antitumor activity of plant-derived non-psychoactive cannabinoids. J. Neuroimmune Pharmacol..

[9] Wong K.Y., Janjua T.I., Martin J.H., Begun J., Popat A. (2025). Cannabidiol Targets Colorectal Cancer Cells via Cannabinoid Receptor 2, Independent of Common Mutations. ACS Pharmacol. Transl. Sci..

[10] Pellerito O., Notaro A., Sabella S., De Blasio A., Vento R., Calvaruso G., Giuliano M. (2014). WIN induces apoptotic cell death in human colon cancer cells through a block of autophagic flux dependent on PPAR c. Apoptosis.

[11] Li D., Ilnytskyy Y., Gojani E.G., Kovalchuk O., Kovalchuk I. (2022). Analysis of Anti-Cancer and Anti-Inflammatory Properties of 25 High-THC Cannabis Extracts. Molecules.

[12] Li S., Li W., Malhi N.K., Huang J., Li Q., Zhou Z., Wang R., Peng J., Yin T., Wang H. (2024). Cannabigerol ( CBG ): A Comprehensive Review of Its Molecular Mechanisms and Therapeutic Potential. Molecules.

[13] Fikrettin Ş. (2023). Cannabinoid compounds in combination with curcumin and piperine display an anti-tumorigenic effect against colon cancer cells. Front. Pharmacol..

[14] Nielsen F.H., Eckhert C.D. (2020). Boron. Adv. Nutr..

[15] Felipe G., Fernandes S., Alexander W., Leandro J., Santos D. (2019). Boron in drug design: Recent advances in the development of new therapeutic agents. Eur. J. Med. Chem..

[16] Frooman M.B., Deb M.K., Peters J., Leggett S., Sanghai N., Rizvi N.Z., Atukorallaya D., Tranmer G.K. (2025). Recent Advancements in the Diversification and Applications of Boron-Containing Compounds in Medicinal Chemistry. Pharmaceuticals.

[17] Mohammed E.E., Türkel N., Yigit U.M., Dalan A.B., Sahin F. (2023). Boron Derivatives Inhibit the Proliferation of Breast Cancer Cells and Affect Tumor-Specific T Cell Activity In Vitro by Distinct Mechanisms. Biol. Trace Elem. Res..

[18] Turkez H., Arslan M.E., Tatar A., Mardinoglu A. (2021). Promising potential of boron compounds against Glioblastoma: In Vitro antioxidant, anti-inflammatory and anticancer studies. Neurochem. Int..

[19] Cebeci E., Yüksel B., Şahin F. (2022). Anti-cancer effect of boron derivatives on small-cell lung cancer. J. Trace Elem. Med. Biol..

[20] Li X., Wang X., Zhang J., Hanagata N., Wang X., Weng Q., Ito A., Bando Y., Golberg D. (2017). Hollow boron nitride nanospheres as boron reservoir for prostate cancer treatment. Nat. Commun..

[21] Yilmaz A.B., Tapsin S., Elbasan E.B., Kayhan H.D., Sahin F., Turkel N. (2019). Suppressor Effects of Sodium Pentaborate Pentahydrate and Pluronic F68 on Adipogenic Differentiation and Fat Accumulation. Biol. Trace Elem. Res..

[22] Demirci S. (2016). Boron promotes streptozotocin-induced diabetic wound healing: Roles in cell proliferation and migration, growth factor expression, and inflammation. Mol. Cell. Biochem..

[23] Sahin F. (2025). Targeting Hippo Signaling Pathway with a Boron Derivative, Sodium Pentaborate Pentahydrate (NaB): Therapeutic Strategies in Colorectal Cancer. Pharmaceuticals.

[24] Ahmed D., Eide P.W., Eilertsen I.A., Danielsen S.A., Eknæs M., Hektoen M., Lind G.E., Lothe R.A. (2013). Epigenetic and genetic features of 24 colon cancer cell lines. Oncogenesis.

[25] Muller P.A.J., Vousden K.H. (2014). Perspective Mutant p53 in Cancer: New Functions and Therapeutic Opportunities. Cancer Cell.

[26] Viereckl M.J., Krutsinger K., Apawu A., Gu J., Cardona B., Barratt D., Han Y. (2022). Cannabidiol and Cannabigerol Inhibit Cholangiocarcinoma Growth In Vitro via Divergent Cell Death Pathways. Biomolecules.

[27] Kletkiewicz H., Wojciechowski M.S., Rogalska J. (2024). Cannabidiol effectively prevents oxidative stress and stabilizes hypoxia-inducible factor-1 alpha (HIF-1α) in an animal model of global hypoxia. Sci. Rep..

[28] Pongking T., Intuyod K., Thongpon P., Thanan R. (2024). Cannabidiol suppresses proliferation and induces cell death, autophagy and senescence in human cholangiocarcinoma cells via the PI3K/AKT/mTOR pathway. J. Tradit. Complement. Med..

[29] Hacioglu C., Kar F., Kacar S., Sahinturk V., Kanbak G. (2019). High Concentrations of Boric Acid Trigger Concentration-Dependent Oxidative Stress, Apoptotic Pathways and Morphological Alterations in DU-145 Human Prostate Cancer Cell Line. Biol. Trace Elem. Res..

[30] Conrad M., Dixon S., Fulda S., Gascon S., Hatzios S.K. (2018). Ferroptosis: A regulated cell death nexus linking metabolism, redox biology, and disease. Ferroptosis.

[31] Yang G., Qian B., He L., Zhang C., Wang J., Qian X., Wang Y. (2025). Application prospects of ferroptosis in colorectal cancer. Cancer Cell Int..

[32] Mahemuti D., Ma L., Siddiqe W., Tang Z., Kong Y., Li W., Zhang Z., Su Z., Maimaitijiang A. (2026). Ferroptosis as a novel therapeutic strategy to overcome multidrug resistance in colorectal cancer. Pharmaceuticals.

[33] Wang Y., Zhang Z., Sun W., Zhang J., Xu Q., Zhou X., Mao L. (2022). Ferroptosis in colorectal cancer: Potential mechanisms and effective therapeutic targets. Biomed. Pharmacother..

[34] Li Y., Bi Y., Li W., Piao Y., Piao J., Wang T., Ren X. (2024). Research progress on ferroptosis in colorectal cancer. Front. Immunol..

