# Supplementary material for: Enhancing Anti-Cancer Efficacy in Colorectal Cancer Through Cannabinoid and Sodium Pentaborate Co-Therapy
Source: Molecules. 2026 Sep 11;31(18):3206. doi: 10.3390/molecules31183206 (PMC13609900; doi:10.3390/molecules31183206)
Supplement: Supplementary file 1 [file molecules-31-03206-s001.zip › molecules-4304747-supplementary materials Figure S1.pdf]

Supplementary Materials

Enhancing Anti-Cancer Efficacy in Colorectal Cancer Through Cannabinoid and Sodium Pentaborate Co-Therapy

Büşra Yüksel, Fikrettin Şahin and Nezaket Türkel *

Department of Genetics and Bioengineering, Faculty of Engineering Natural Science, Yeditepe University, Kayışdağı, Istanbul 34755, Turkey; busra.yuksel@yeditepe.edu.tr (B.Y.); fsahin@yeditepe.edu.tr (F.Ş.)

* Correspondence: nezaket.turkel@gmail.com

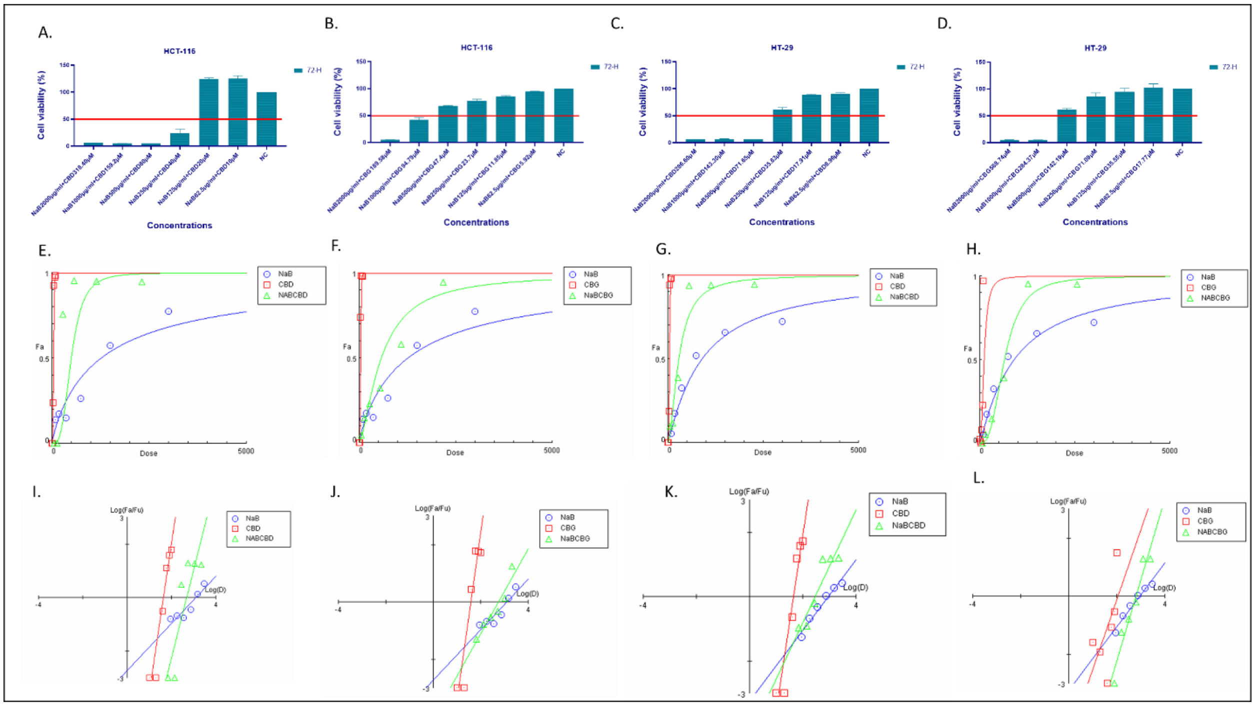

Supplementary Figure S1: Fixed-ratio combination analysis of NaB with CBD or CBG in HCT-116 and HT-29 cells. (A–D) Cell viability after 72 h treatment. Data are shown as mean ± SD (n = 3). The red line represents 50% viability. (E–H) CompuSyn dose–effect curves. (I–L) Median-effect plots generated according to the Chou–Talalay method.
